# Sclerotinic Acid in Hæmoptysis

**Published:** 1879-09

**Authors:** 


Sclerotinic Acid in Hæmoptysis.—This acid, obtained first by Dragendorff from ergot, has lately been adopted by von Ziemssen and other German physicians as preferable to ergotin for hypodermic injection in hæmoptysis and other internal hemorrhage. A five per cent. solution is used, and it is said not to be so liable to be followed by abscesses as ergotin.—*Med. and Surg. Rep.*

